# Cross-cultural adaptation of the *Parental Perceptions of Children’s Exposure to Tobacco Smoke* instrument to the Brazilian context

**DOI:** 10.1186/s13104-023-06513-w

**Published:** 2023-09-26

**Authors:** Maria Alice Santos Tavares, Vicki Myers, Leandro Alberto Calazans Nogueira, Agnaldo José Lopes

**Affiliations:** 1 Post-Graduation Programme in Rehabilitation Sciences, Centro Universitário Augusto Motta (UNISUAM), Bonsucesso, Rio de Janeiro, 94, 21032-060 Brazil; 2 Instituto Nacional do Câncer José Gomes de Alencar, Rio de Janeiro, Brazil; 3 Department of Health Promotion, School of Public Health, Sackler Faculty of Medicine, Tel Aviv University, Tel Aviv, Israel; 4 Gertner Institute of Epidemiology & Health Policy Research, Sheba Medical Center, Ramat Gan, Israel; 5 Physiotherapy Department, Instituto Federal do Rio de Janeiro (IFRJ), Rio de Janeiro, Brazil; 6 Post-Graduation Programme in Medical Sciences, School of Medical Sciences, Universidade do Estado do Rio de Janeiro (UERJ), Rio de Janeiro, Brazil

**Keywords:** Validation study, Tobacco smoke pollution, Child, Perception

## Abstract

**Objective:**

To perform a cross-cultural adaptation of the *Parental Perceptions of Children’s Exposure to Tobacco Smoke* (PPE) instrument to the Brazilian context.

**Results:**

The cross-cultural adaptation process was performed in 10 stages. Four translators, eight specialists, and 35 primary care users participated in the study. Both translations were similar. The synthesis version that was back-translated was equivalent to the original. The committee of experts scored all items in the content validity index as 3 or 4, suggesting only small changes such as changing “photo” to “image” and “service balcony” to “service area”. After the completion of the first sequence of pretests, some adjustments were required by the committee of experts for the second round. The form of application of the self-administered questionnaire for the interview was changed, the Likert scale was reduced from 7 to 5 points, and the option “I don’t know” was added to questions 18, 19, and 20. After these adjustments, the instrument was well accepted by the study population and presented good internal consistency (Cronbach’s α score = 0.82). The PPE instrument, which assesses the perception of parents about their children’s exposure to cigarette smoke, was satisfactorily translated and adapted to the Brazilian context.

## Introduction

The World Health Organization warns that half of children worldwide are exposed to tobacco smoke, and approximately 65,000 children die annually as a result of diseases related to passive smoking [1]. In Brazil, an estimated 15 million children are passive smokers [2]. Parent’s reports of their children’s exposure to tobacco smoke depends on parental information, which can often be inaccurate or underreported. Possible explanations for this situation include parental denial of exposure, social desirability bias, recall bias, and mis-understanding of what exposure is [3]. In addition, the attitude of parents to smoke around their children is influenced by people’s perception of tobacco exposure. Some people understand that exposure occurs only when smoke can be seen or smelled, although others have a broader understanding and understand that exposure exists even in the absence of these factors and even if the smoker is in an open and/or ventilated environment [4].

The instrument *Parental Perceptions of Children’s Exposure to Tobacco Smoke* (PPE) aims to measure the perception of parents about the exposure of children to tobacco smoke [5]. The instrument was produced from a qualitative study with 65 Israeli smoking parents through interviews about exposure to tobacco smoke, and validated in a survey with 220 parents [4, 5].

After the analysis, two general concepts emerged about the parents’ perceptions of exposure: (1) sensory perceptions (smell and vision) and (2) physical context (proximity, space, movement, and time). Next, the instrument was designed with six aspects: (1) second-hand exposure, (2) third-hand exposure, (3) knowledge/perceived certainty, (4) sensory perceptions, (5) perceptions of time, and (6) perceptions of distance. Therefore, the instrument was designed to represent each of the aspects involving exposure to tobacco smoke and to quantify the parents’ perceptions of their children’s exposure to cigarette smoke. The instrument uses photos of children in carious places with people smoking, and parents are asked to rate to what extent they think the child is exposed on a scale. These pictures were used to anchor the question, so that the situations were less open to interpretation and everyone would be relating to the same circumstances.

The process of cross-cultural adaptation is necessary when measuring health issues in a population using a construct developed in another cultural setting [6]. This process allows studies to be conducted more quickly and more economically, in addition to allowing results to be compared among populations in different countries and cultures [7]. In this context, the present study aimed to describe the process of translation and cross-cultural adaptation of the PPE instrument into Portuguese spoken in Brazil.

## Main text

### Patients

**Fig. 1:**
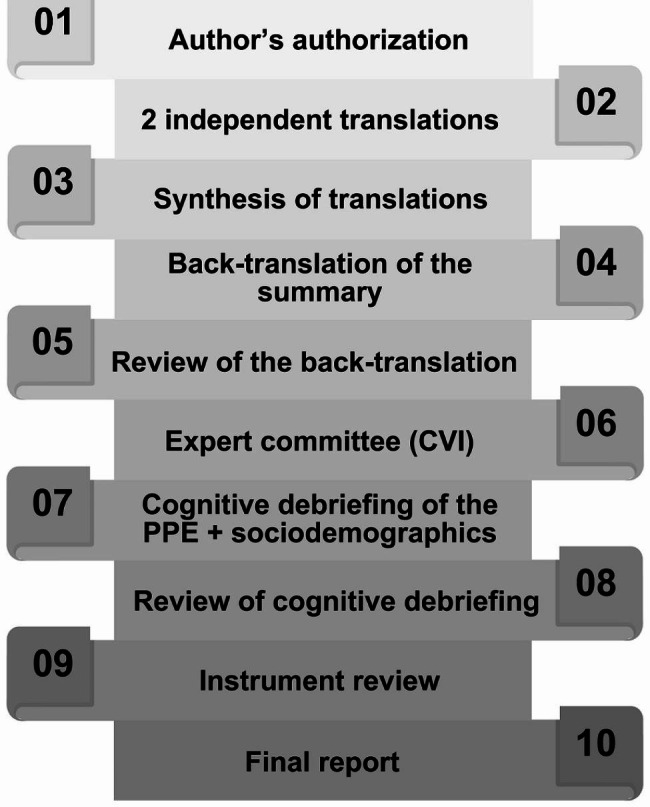
Flowchart showing the 10 stages of the study. *CVI* content validity index, *PPE Parental Perceptions of Children’s Exposure to Tobacco Smoke*

A cross-cultural adaptation study was conducted following the guidelines of Wild et al. [8] and consisted of 10 stages, as shown in Fig. 1. The same pictures of the original instrument were used in our study. The protocol was approved by the Research Ethics Committee of the Municipal Department of Health and Civil Defense of Rio de Janeiro under number CAAE:55824922.6.00005279, complying with the ethical principles of research on human beings established by Resolution No. 466/2012 and the Declaration of Helsinki. After authorization by the authors of the instrument by electronic communication in March 2021, the study was conducted between April 2021 and January 2023.

### Parental Perceptions of Children’s Exposure to Tobacco Smoke

After the authors’ authorization (Stage 1), two bilingual translators performed independent translations (Stage 2) of the source instrument into Brazilian Portuguese. One translator was a health care professional with experience in cross-cultural adaptation and knowledge of the objectives of the instrument (clinical translator, T1); the other was blind (naive translator, T2). Next (Stage 3), a synthesis of the translations was conducted (T1-2) with the presence of both translators, the researcher, and a bilingual native Brazilian who did not participate in the previous stage and was knowledgeable about the research objectives. The T1-2 version was back-translated (Stage 4) by two other professional bilingual translators (R1-R2) without knowledge of the instrument. A review of the back translations (Stage 5) did not identify discrepancies from the original.

A committee (Stage 6) composed of 8 experts (1 epidemiologist, 2 linguists, 3 nurses with experience in cross-cultural adaptation, and 2 bilingual laypeople from the target population) evaluated the semantic, idiomatic, experimental, and conceptual equivalence. The committee members scored the questions by marking the following options: 1 = very unclear, 2 = unclear, 3 = clear, and 4 = very clear. These members were instructed to suggest changes. Consensus among the experts was established by calculating the content validity index (CVI) reached by counting the number of responses scored as 3 or 4 and dividing by the total number of responses, with a value above 0.9 considered acceptable.

The first pretest version of the PPE instrument in Portuguese was obtained. The original version in English has 23 items, 20 of which are scored on a 7-point Likert scale ranging from 1 (“no exposure”) to 7 (“highly exposed”) and 3 with open-ended responses. The Brazilian version of the instrument transformed the open responses into closed responses following the suggestions of the authors of the original study due to the low reliability of temporal stability in the test-retest of the instrument with open responses. The sum of the PPE was obtained by adding all the scores of the items, while the mean score was obtained by dividing the total sum by the number of answered items. This version was applied in the cognitive debriefing (Stage 7), which aims to assess the acceptability, understanding, and clarity of the instrument items by the target population and to observe the completion time. After each question, the participants were asked to rate each item in terms of their understanding as good (they had no doubts with the question and/or answer), fair (there was some uncertainty with the question and/or answer), or poor (they did not understand any part of the question and/or answer). In addition to the PPE, a questionnaire was applied to collect sociodemographic data. Parents who were users of primary care in the area of the Coordination of Program Area 5.3 in the city of Rio de Janeiro, of both sexes, smokers or not, with children up to 5 years of age were recruited by convenience to fill out the instrument. Parents with some intellectual disability were excluded from the study. The participants were informed about the objectives of the study, and after signing the informed consent form, they were asked to answer the questionnaire individually.

After the application of the first version of the pretest for five participants, some questions had their understanding considered as regular. Furthermore, the participants who had not completed the fundamental school expressed difficulty in differentiating perception from exposure on a scale of 1 to 7, showing a longer time to finish the instrument. Before that, it was necessary for a new meeting with the committee of experts to, consensually, discuss health literacy, the best form of application of the instrument, and the feasibility of reducing the scale rating without harming internal consistency.

Stage 8 consisted of reviewing the results of the cognitive debriefing followed by a review of the instrument (Stage 9) in search of minor errors and preparation of a final report (Stage 10).

## Results

The translation stage was completed with similar versions. The clinical translator developed an informal translation more suited to the audience in question, while the naive translator produced a document with more formal language. The divergent expressions were analyzed together with the translators and the researcher, resulting in the synthesis version of the PPE for Portuguese spoken in Brazil, which is presented in Table 1.

**Table 1:** Synthesis version of the *Parental Perceptions of Children’s Exposure to Tobacco Smoke* into Portuguese spoken in Brazil

Original	Translator 1	Translator 2	Synthesis T1/2
Parental perceptions of exposure questionnaire	Percepção dos pais sobre o questionário de exposição	Percepção dos pais sobre o questionário de exposição	Percepção dos pais sobre o questionário de exposição
A. In this questionnaire, you will be presented with various situations showing smokers and children.	Neste questionário você será apresentado à várias situações mostrando fumantes e crianças.	Neste questionário você será apresentado à diferentes situações contendo fumantes e crianças.	Neste questionário você será apresentado à várias situações mostrando fumantes e crianças.
To what degree do you think the child in the picture is exposed to cigarette smoke? (To what degree does the smoke reach him/her?). Rate your answer from 1 = not at all to 7 = highly.	A que grau você acredita que a criança na foto está sendo exposta ao fumo de cigarros? Marque sua resposta de 1 (de jeito nenhum) até 7 (altamente exposta).	Em qual grau você acha que a criança na imagem está exposta à fumaça do cigarro? Classifique sua resposta entre 1 (nenhuma exposição) e 7 (altamente exposta).	Em qual grau você acha que a criança na imagem está exposta à fumaça do cigarro? Classifique sua resposta entre 1 (nenhuma exposição) e 7 (altamente exposta).
B. In the following questions, situations will be described without pictures. Please rate to what degree you think the child described is exposed to cigarette smoke? (To what degree does the smoke reach him/her?)	Nas perguntas seguintes as situações serão descritas sem fotos. Por favor, marque o grau que você pensa que as crianças descritas estão expostas ao fumo do cigarro? (a que grau a fumaça o/a alcançar?)	Nas próximas questões serão descritas situações sem fotos. Avalie, em sua opinião, o grau de exposição da criança à fumaça do cigarro. (Em qual grau a fumaça atinge a criança)	Nas perguntas seguintes as situações serão descritas sem imagens. Por favor, avalie, em sua opinião, o grau de exposição da criança descrita a fumaça do cigarro. (Em qual grau a fumaça atinge a criança)
9. The child is in the kitchen, and someone is smoking on the adjacent service balcony.	A criança está na cozinha, alguém está fumando na varanda de serviço ao lado	A criança está na cozinha e há alguém fumando na varanda adjacente.	A criança está na cozinha, alguém está fumando na área de serviço ao lado.
10. The child is in a room where someone smoked 12 h ago.	A criança está em um quarto onde alguém fumou 12 h atrás.	A criança está em um cômodo onde alguém fumou 12 h atrás.	A criança está em um quarto onde alguém fumou 12 h atrás.
11. The child is in a room where someone smoked 2 h ago.	A criança está em um quarto onde alguém fumou 2 h atrás.	A criança está em um cômodo onde alguém fumou 2 h atrás.	A criança está em um quarto onde alguém fumou 2 h atrás.
12. The child is in a room where someone smoked 30 min ago.	A criança está em um quarto onde alguém fumou 30 min atrás.	A criança está em um cômodo onde alguém fumou há 30 min.	A criança está em um quarto onde alguém fumou 30 min atrás.
13. The child is in a car where someone smoked 1 h ago	A criança está em um carro onde alguém fumou 1 h atrás.	. A criança está em um carro onde alguém fumou 1 h atrás.	A criança está em um carro onde alguém fumou 1 h atrás.
14. The child is in a car where someone smoked 20 min ago	A criança está em um carro onde alguém fumou 20 min atrás.	A criança está em um carro onde alguém fumou há 20 min.	A criança está em um carro onde alguém fumou 20 min atrás.
15. The child is in the playground and sees his mother smoking but does not smell smoke.	A criança está no parquinho e vê a sua mãe fumando, mas não sente o cheiro da fumaça.	A criança está em um playground e vê a mãe fumando, mas não sente o cheiro da fumaça	A criança está no parquinho e vê a sua mãe fumando, mas não sente o cheiro da fumaça
16. The child is in the playground and smells cigarette smoke but does not see the smoker.	A criança está no parquinho e sente o cheiro da fumaça do cigarro, mas não vê o fumante.	A criança está em um playground e sente o cheiro da fumaça do cigarro, mas não vê o fumante.	A criança está no parquinho e sente o cheiro da fumaça do cigarro, mas não vê o fumante.
17. The child is in the playground and sees his mother smoking and can smell the smoke.	A criança está no parquinho e vê a sua mãe fumando e consegue sentir o cheiro da fumaça.	A criança está em um playground, vê a mãe fumando e sente o cheiro da fumaça.	A criança está no parquinho e vê a sua mãe fumando e consegue sentir o cheiro da fumaça.
18. What proportion of tobacco smoke is invisible?	Qual a proporção da fumaça do tabaco é invisível?	Qual a fração da fumaça de cigarro não é visível?	Qual a proporção da fumaça do tabaco é invisível?
19. After smoking in the home, how long does it take for the home to be free of smoke?	Depois de fumar em casa, quanto tempo leva para a casa ficar livre de fumaça?	Após uma pessoa fumar em casa, quanto tempo leva para a casa estar livre da fumaça? Resposta livre.	Depois de fumar em casa, quanto tempo leva para a casa ficar livre de fumaça?
20. After smoking in the car, how long does it take for the car to be free of smoke?	Depois de fumar no carro, quanto tempo leva para o carro ficar livre de fumaça?	Após uma pessoa fumar em um carro, quanto tempo leva até o carro estar livre de fumaça? Resposta livre	Depois de fumar no carro, quanto tempo leva para o carro ficar livre de fumaça?
21. Do you consider yourself to have sufficient information on the subject of passive smoking?	Você se considera suficientemente bem informado sobre o fumo passivo? (de jeito nenhum - muito)	. Você considera que sabe o suficiente sobre o assunto “fumante passivo”.	Você considera que sabe o suficiente sobre o assunto “fumante passivo”.
22. How confident do you feel about your answers?	Quão confiante você se sente com as suas respostas?	O quão confiante você se sentiu ao responder o formulário?	Quão confiante você se sente com as suas respostas?
23. Did you find it difficult to answer the questionnaire?	Você teve dificuldade para responder este questionário?	Você achou difícil responder ao questionário?	Você achou difícil responder ao questionário?

The greatest divergence between versions T1 and T2 referred to the term “room” from questions 10, 11, and 12, translated by the clinical translator as “quarto” and by the naive translator as “cômodo”. At the meeting, the term “quarto” was chosen because other rooms in the house were already described in the instrument. In terms of the definition of the terms of the Likert scale, the term “1 - no exposure” was chosen for the naive translator instead of “1 - not at all” for the clinical translator.

The synthesis version was back-translated by two professional translators who did not participate in the previous stage and who were blinded to the objectives; this version was equivalent to the original, corroborating that the process was satisfactory until this stage. The committee of experts evaluated the semantic, idiomatic, experimental, and conceptual equivalence using the CVI calculation. Although all CVI items scored 3 or 4, after a second round of analysis by the committee, it was convenient to replace the term “service balcony” in question 9 with “service area” more common to the Brazilian population, as well as the word “photo” in statement B with “image” because it is a more impersonal word.

In the initial pretest, five participants were asked to fill out the questionnaire without assistance. The understanding of items 18, 19, and 20, which covered knowledge about third-hand exposure, was rated as fair by 60% of participants. It was observed that participants who had less education (i.e., incomplete elementary school) had some difficulty in relating the items with the 7-point Likert scale. This group also took a longer time completing the instrument (mean time 17 min) compared to participants who had completed elementary school, high school, and/or higher education (mean time 11 min).

After a new meeting of the committee, the second pretest version was applied in the form of an interview with a 5-point Likert scale based on the difficulties presented by the participants who did not finish elementary school and the inclusion of the option “I don’t know” in Items 18, 19, and 20. In this second round of the pretest, 30 parents participated. The majority were mothers (77%), with a mean age of 34.2 ± 11 years who had completed elementary school (43%). The mean number of children was two, ranging from 1 to 5, and 86% of the participants declared themselves to be nonsmokers or former smokers, although 36% of them reported living in a household with a smoker. The understanding of all the questions was rated as good by the participants, especially for participants with low education, indicating that the new response format with 5 items was better accepted. In addition, there was a reduction in the average completion time from 17 min to 7.6 min for participants who had not completed elementary school and from 11 min to 5.8 min for those who had completed high school or higher education.

The two sets of scores were subjected to a normality analysis using the Shapiro-Wilk test. The test result indicated a *P*-value of 0.569 for the PPE scores on a 5-point Likert scale and a *P*-value of 0.223 the PPE scores on a 7-point Likert scale. Therefore, it is considered that both scores have a normal distribution. Subsequently, the Student’s *t* test was performed for independent samples, which did not show a statistically significant difference (*P* = 0.065) between the two versions of pretests, indicating that this change did not affect the results of the study.

The mean PPE score was 3.93 ± 0.48, ranging from 3.00 to 4.80. The mean sum of the instrument was 78.63 ± 9.77 points, ranging from 60 to 96 points. The value of Cronbach’s α was 0.82.

## Discussion

The objective of this study was to perform a cross-cultural adaptation of the PPE to the Brazilian context. After the changes suggested by the expert committee, all participants in the second round of the pretest reported that they understood all items of the questionnaire, which indicates that the process was successful. Furthermore, the instrument exhibited good internal consistency, as the Cronbach’s α value was between 0.70 and 0.95 [9].

Considering the impact that passive smoking causes on society, especially when it involves children, who have no option to expose themselves or not, the instrument adapted in this study may be useful in Brazil because, especially with regard to educational issues, understanding how parents perceive exposure to passive smoking can help health professionals direct the information provided. In addition, the instrument can correct mistakes, make parents aware of exposure in various circumstances, and help protect their children [5, 10].

The pretest was performed to identify possible problems in the application and understanding of the instrument. A question that emerged during this stage was about which method would be the most favorable for application of the instrument. With this in mind, an “interview” or “self-completion” was considered, taking into account the discrepancy in the participants’ education. To minimize this problem, the committee met for a debate based on the study by Coelho and Esteves [11], who emphasize that respondents who are more skilled and experienced in the response scale allow the use of a greater number of options and that 5-point scale has an adequate level of reliability, adjusting to respondents of different skill levels. Corroborating this fact, studies conducted by Dalmoro and Vieira [12] indicate that 7 points is the limit of the human capacity to discern and make judgments, as demonstrated by participants who dominate the subject and have experience in answering scales. It is noteworthy that in the initial study, cross-validation was carried out by comparing the original instrument with a risk perception scale composed of 17 items, 8 of which were images and 9 were text, scored on a 7-point Likert scale, following the same format as the PPE. [5]. A study carried out in Malaysia adapted and used the image section of the risk perception scale, where it was also essential to reduce the Likert scale to 5 points [10]. The results obtained were similar, and showed that smoking parents have lower levels of risk perception than non-smoking parents.

Considering that health literacy refers to the level of understanding of essential information to make decisions in the field of health and that the health team must recognize that low levels of health literacy demand greater performance, especially in the competencies of communication, a new debate was held [13–15]. With the approval of the instrument’s authors, it was decided to perform the pretest in the form of an interview with a 5-point Likert scale and to include the option “I don’t know” in questions 18, 19, and 20, which were classified as regular, not adequate, for understanding the question, not because the question was paraphrased improperly but because of a lack of knowledge of the answer.

Although changes were made in the form of application and in the score of the scale for its use in Brazil, the process of translation and cross-cultural adaptation showed good results, and the CVI was satisfactory. The detailed description of all stages may be reproduced in other processes of translation and cross-cultural adaptation of the original instrument. In the original study,^5^ the instrument was designed for self-application. However, the level of education of the participants was not reported. In our study, relative difficulty in understanding the questions was observed, especially in those with lower educational levels.

In conclusion, the results indicate that the Brazilian version of the PPE seems to be promising when applied in an interview format with a 5-point Likert scale. Therefore, psychometric studies for the analysis of reliability and validity should be performed to complement the process of translation and cross-cultural adaptation of the version of the PPE into Portuguese spoken in Brazil.

### Limitations

Some limitations of our study should be noted. First, the sample size is small and, therefore, the results obtained should be evaluated with caution. Second, the scale was not subjected to the process of translation and adaptation in other cultures, which makes it impossible to debate and compare the results. Finally, it is necessary to validate the measurement properties by evaluating a more robust sample of the translated version of the scale. In this next stage of the research, it is essential to use the validated questionnaire.

## Data Availability

All the data supporting the results are provided in the manuscript.

## Abbreviations

CVI: Content validity index
PPE: *Parental Perceptions of Children’s Exposure to Tobacco Smoke*
T1: Clinical translator
T2: Naive translator
